# Science: a world without borders

**DOI:** 10.1007/s13238-015-0189-6

**Published:** 2015-07-12

**Authors:** Joel Haywood

**Affiliations:** CAS Key Laboratory of Pathogenic Microbiology and Immunology, Institute of Microbiology, Chinese Academy of Sciences, Beijing, 100101 China

In 2010 the effects of the global financial crisis were still being felt in the UK. Stock market confidence levels were low, unemployment levels were high and job opportunities were few and far between especially for new graduates with little work experience. Unfortunately for my partner, her recent graduation and search for job opportunities within the finance and law sector could not have come at a more unsuitable time. China however had better weathered the storm of the financial crisis and presented itself as a more familiar and fruitful climate for my partner to further develop her career. Furthermore, the rising media coverage of China’s outstanding growth and its predicted future importance in world economic and scientific affairs had aroused my inquisitive nature, especially as I understood so little of its culture. Therefore, in 2010 we decided to move to China together and we were fortunate enough to quickly find opportunities for us in Beijing; my partner as a lawyer in a leading law firm and myself enrolled in a joint-master Ph.D. scholarship funded by the Chinese Academy of Sciences.

Upon commencement of the Ph.D. program led by the Chinese Academy of Sciences all students participate in a Chinese cultural awareness course held in Beijing for five months. During this time students are taught the basics of the Chinese language and cultural history. This experience was the first of many truly rewarding moments of my time in China. This was my first experience of being an international student and, through sharing classes and free time, provided me with the chance to form lasting friendships with students from a diverse range of nations including: America, D. P. R. Korea, R. O. Korea, Kazakhstan, Nepal, India, Pakistan, Iran, Uganda, Nigeria and Kenya. Moreover, I also came to appreciate our cultural differences and similarities and became increasingly conscience of how, despite our geographical and cultural environments, we all shared the same desire to explore utilize science for the betterment of humanity. Those five months have left a lasting imprint on my memory of how I furthered my appreciation for different world cultures and widened my outlook on the international scientific research community.

Following the completion of our Chinese cultural awareness course the years Ph.D. students departed to their respective institutes. Personally this entailed the short trip to the Institute of Microbiology, Beijing, as I was fortunate enough to have been accepted for Ph.D. supervision by one of China’s most eminent researchers, Professor George Fu Gao. Prior to undertaking a Ph.D. in China I had previously worked for a small university spin-out company from the University of Southampton and had participated in Phase I and II clinical trials and viral infection research focused upon asthmatics and COPD patients (Xiao et al., 2011). In this industrial research environment I developed a passion for translational research focused on new and emerging viral infections. By undertaking a Ph.D. under Professor George Fu Gao’s supervision I hoped to further my knowledge of a molecular understanding of viral infections and the immune response. Moreover, Professor George Fu Gao’s previous international studies experience at Harvard and Oxford universities combined with his prolific rate of high quality scientific publication rate encouraged me to believe that the Chinese Academy of Sciences could provide a fertile environment for me to undertake scientific research.

This assumption has been fulfilled throughout conduction of my research at the Institute of Microbiology, with Professor George Fu Gao fostering a challenging, innovative and international research environment at the Chinese Academy of Sciences. Obviously, there are difficulties to be overcome with working in a multicultural environment. Personally, I also needed to adjust to carrying out research in academic setting with new policies and procedures. However, these challenges provide new opportunities for individuals to share and benefit from new practices and ideals, developing the individual’s skills of adaptability, flexibility and cultural sensitivity as well as the group’s scientific productivity. By facing these challenges in a positive manner and by constantly striving for an improvement in scientific research quality Professor George Fu Gao’s laboratory has been able to make a vast array of important scientific discoveries which have been published in highly regarded scientific journals. Fortunately for me, I was able to be part of many of these discoveries and have benefitted from the experience of working on collaborative research projects besides my own research project (Cui et al., 2014; Shi et al., 2014; Tefsen et al., 2014; Shi et al., 2013). Moreover, I have been given the opportunity to attend many international scientific meetings and discussions that have aided my understanding of research directions at the forefront of scientific research.

Now my Chinese journey is nearly complete. I will soon graduate from the Chinese Academy of Sciences and after recently marrying my partner; we have decided to continue the next stage of our life in another country. Fortunately for me, as a native English speaker, science is a world without borders. The experience of carrying out international studies in China has been an invaluable one. I now have the confidence to approach any research environment with skills and novel approaches to problem solving that I may never have developed had a chosen to continue my further education in my homeland. I would strongly recommend carrying out international studies to any students contemplating such an adventure and encourage non-native English language speakers continue their efforts studying English in order to increase their opportunities for studying and working in the international scientific community. Moreover, I hope that the Chinese Academy of Sciences and other universities can continue to encourage and further investment in student exchange programs and in the opportunities given for students to attend international meetings.

In uncertain economic climates, with a backdrop of austerity, fear and protectionism can all too easily creep into a nation’s mindset. However, in these globalized times communication, collaboration and an understanding of cultural awareness are key to combating the problems of disease control that are associated with the expansion in global travel. Moreover, the control of localized outbreaks of disease requires diligent assessment and surveillance in order prevent global spread, as exemplified by the recent outbreaks of Ebola, Influenza and MERS. We must therefore strive to be more altruistic, as by increasing the opportunities for international collaboration and communication of scientific research we will undoubtedly enhance our abilities in disease prevention and control for the betterment of all mankind.
